# Can Neural Networks Learn Atomic Stick–Slip Friction?

**DOI:** 10.1021/acsami.5c09866

**Published:** 2025-07-09

**Authors:** Mahboubeh Shabani, Andrea Silva, Franco Pellegrini, Jin Wang, Renato Buzio, Andrea Gerbi, Andrea Vanossi, Ali Sadeghi, Erio Tosatti

**Affiliations:** † Department of Physics, 422997 Shahid Beheshti University, 1983969411 Tehran, Iran; ‡ CNR-IOM, Consiglio Nazionale delle Ricerche - Istituto Officina dei Materiali, c/o SISSA, Via Bonomea 265, 34136 Trieste, Italy; § 19040 International School for Advanced Studies (SISSA), Via Bonomea 265, 34136 Trieste, Italy; ∥ CNR-SPIN, Consiglio Nazionale delle Ricerche - Istituto Superconduttori, Materiali Innovativi e Dispositivi, C.so F.M. Perrone 24, 16152 Genova, Italy; ⊥ International Centre for Theoretical Physics (ICTP), Strada Costiera 11, 34151 Trieste, Italy

**Keywords:** machine learning, nonlinear friction, atomic stick−slip, neural network, nano tribology

## Abstract

Nanofriction experiments typically produce force traces exhibiting atomic stick–slip oscillations, which researchers have traditionally analyzed with ad hoc algorithms. This study successfully unravels the potential of machine learning (ML) to interpret nanofriction force traces and automatically extract Prandtl–Tomlinson (PT) model parameters. A prototypical neural network (NN) perceptron was trained on synthetic force traces generated by simulations across a wide parameter range. Despite its simplicity, this NN successfully analyzed experimental data, marking the first application of a network trained solely on computational data to experimental nanofriction. Challenges encountered in developing the NN model proved to be instructive and revealing. Poor transferability from synthetic to experimental data sets was resolved by incorporating physics-based descriptors into the synthetic training data, without experimental input. Our protocol’s simplicity underscores its proof-of-concept nature, paving the way for advanced approaches. Validation with experimental data, such as graphene-coated AFM tips on 2D materials, highlights the promise of this ML approach for stick–slip nanofriction studies.

## Graphical abstract

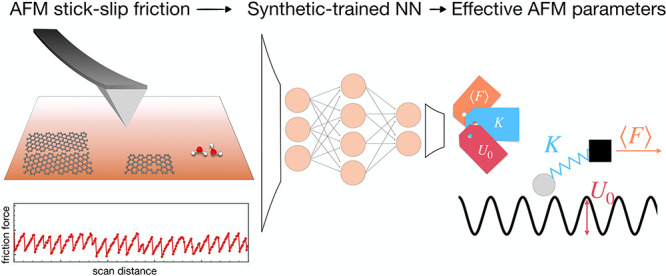

## Introduction

Friction plays an important role in our daily life. A ubiquitous dry friction phenomenon is stick–slip, observed from nanoscale contact shearing to geophysical scale earthquakes. This nonequilibrium process, resulting from a mechanical instability first described by Prandtl^1,2^ is generally highly nonlinear, characterized by long periods of quiet stress accumulation (sticking) followed by sudden and severe energy dissipation events (slips).^3–5^ Unfortunately for theorists, the frictional shear between solids, even in its simplest form without lubricants or wear, is physically complex, involving too many parameters. These aspects, nonequilibrium, nonlinearity, and complexity, make it challenging to describe friction with the tools of statistical physics and to link experimental measures and theoretical predictions.

The usefulness of machine learning (ML) is currently emerging across all disciplines, including some recent applications to tribology.^6–12^ In this work, we show that ML can be harnessed to partly tame and bypass the complexity of stick–slip friction. The starting point is the recent simulation^13^ and experimental^14^ work suggesting that for a given velocity and temperature, the frictional behavior of even relatively large meso- and microscale sliders can be quantitatively described by four parameters of an effective Prandtl-Tomlinson (PT) point-slider model. In that historical model, so far only validated for nanoscale sliders,^15^ a point mass *m* is forced by a spring *K* to slide over a sinusoidal potential of amplitude *U*_0_ (the barrier), the frictional work absorbed by a damping γ. As a general functional interpolator, ML appears ideally suited to the task of automatically interpreting experimental frictional data in the framework of the PT model, a heavy procedure now carried out with laborious algorithms by experimentalists.^14^

Here, we therefore address the basic question: can ML capture the physics of nanoscale sliding and yield a quantitative prediction for the key PT parameters that control atomic stick–slip frictional dissipation? In particular, we shall focus on Atomic Force Microscopy (AFM) experiments based on the relative sliding of 2D material flakes, as sketched in Figure 1.

**Figure 1:**
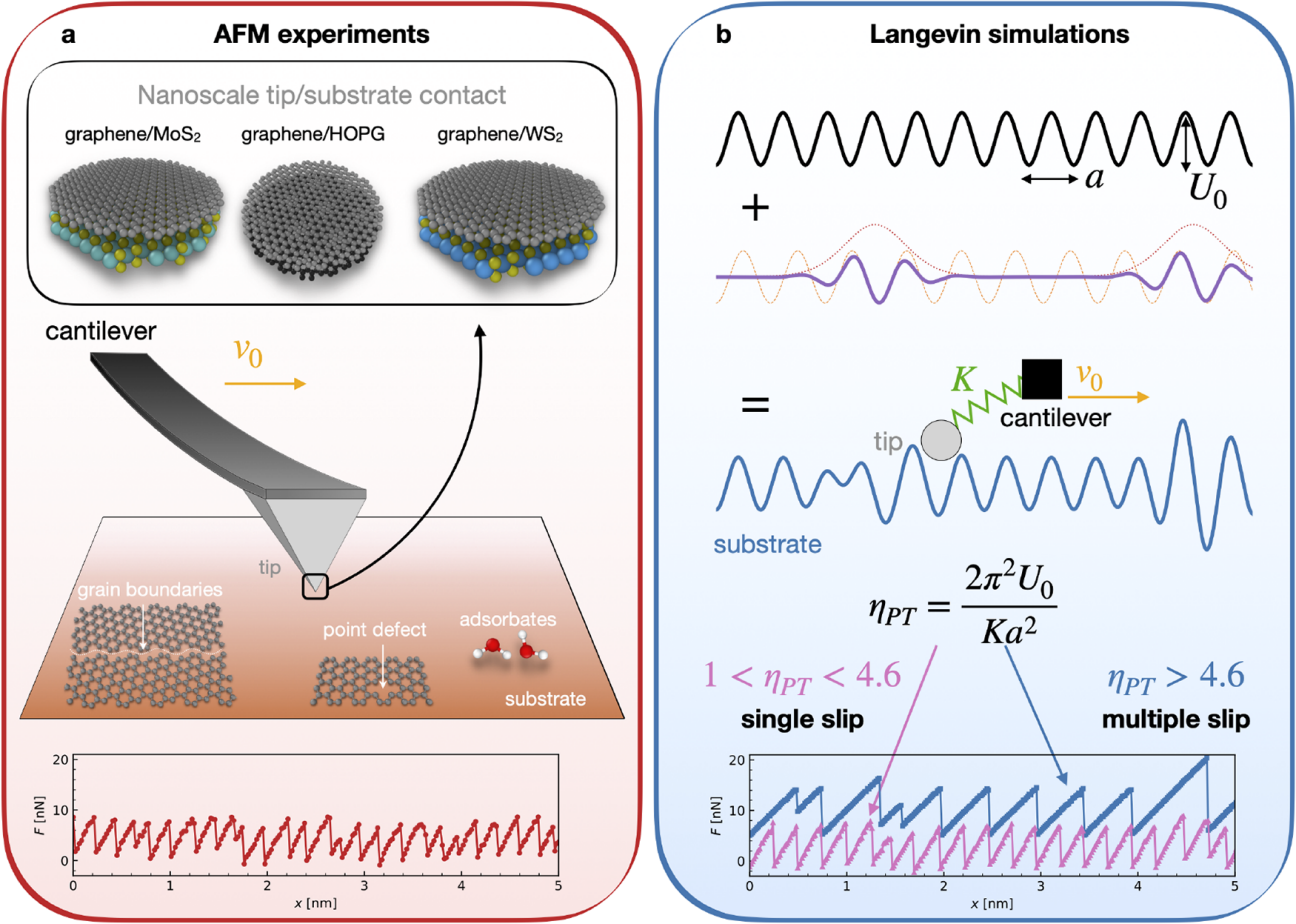
Experimental setup and physical model. (a) In the main middle panel, sketch of a typical AFM experiment highlighting the moving cantilever and the tip sliding over a substrate at constant speed *v*_0_. A set of defects like grain boundaries, point defects, and adsorbates, is shown. The upper inset schematizes the nanoscale contacts realized in our experimental setup, where 2D materials are slid against each other. The bottom panel reports a representative experimental force trace recorded as the cantilever slides at constant speed *v*_0_ over the surface. (b) Sketch of the augmented PT model deployed in this study. The model is composed of the standard sinusoidal modulation of height *U*_0_ and spacing *a* augmented with a localized distortion emulating the defects present on the real surface. The resulting substrate is depicted in blue, along with the modeled AFM point-like tip attached to a translating cantilever by an effective spring of constant *K*. The sliding behavior of the model is determined by the dimensionless parameter η_PT_. The simulated force traces relative to the single and multiple slips regimes are reported at the bottom of panel b in pink triangles and blue squares, respectively.

Compared to the energy landscape of an ideal PT model (see Figure 1b), the interface in real AFM measurements is complicated by the extended size of the frictional contact, of generally unknown atomic structure and shape, as well as by surface defects including grain boundaries, point defects, and adsorbate molecules, as sketched in Figure 1a. The friction of extended sliding contacts is also influenced by edges, which constitute omnipresent defects.^5,13,16^ In order to capture the essential PT parameters out of this complex energy landscape, we augment the standard PT model sinusoidal potential structure with a long-wavelength modulation mimicking the structural defect and complexity of the real surface.^17,18^ The practical goal of this work is to analyze raw force traces—such as the experimental and simulated examples shown at the bottom of Figure 1a,b, respectively—and assess whether a Neural Network (NN) can learn the underlying physics of the PT model, including its effective parameters, directly from these unprocessed data. Importantly, no explicit information about the PT model is hard-coded into the NN architecture. The network is trained only on synthetic raw traces paired with their corresponding energy barrier and local stiffness values. It is then the NN’s task—without prior knowledge of the PT model—to infer the atomic stick–slip laws by identifying patterns and correlations within the input data, and subsequently transfer this learned representation from synthetic (PT-generated) data to experimental data, which is not inherently governed by the PT model. Successful extrapolation would suggest that the PT model offers a meaningful framework for interpreting the experimental system; on the other hand, failure to generalize would indicate that the experimental scenario involves physical complexities beyond the scope of the PT model.

Creating by simulations a curated data set with the relevant sliding physics is the first step in our workflow, as shown in Figure 2a. This data set is used to train a NN via supervised learning: a fraction of the raw trajectories is fed to the NN model along with the labels, while the remainder is left for validation. As we discuss below, this training yields very reliable results. The next, more ambitious goal is to then use this model, now trained solely on synthetic data (like the one at the bottom of Figure 1b), to identify the best PT model parameters behind all new, raw experimental friction force traces (like the one at the bottom of Figure 1a). Note that while the training data are generated by a simulated 1D model, the experimental data come from the vastly more complex 2D interfaces between 3D solids. The PT model used is, of course, unable to capture this complexity. However, as we shall see, embedding in the model a few simple additional details makes the problem learnable by the NN, and the protocol viable.

**Figure 2:**
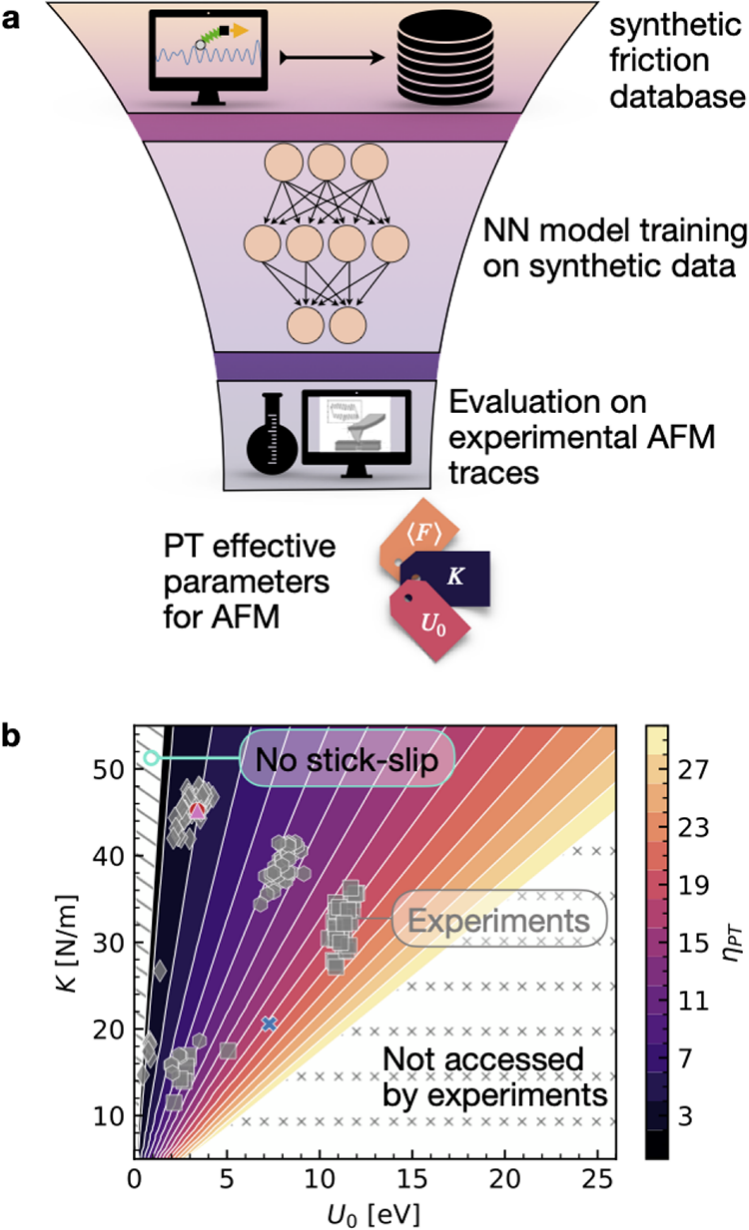
Workflow and system parameter space. (a) Sketch representing the workflow developed in this study. Starting at the top, a systematic exploration of the parameter space via Langevin simulations of the augmented PT model yields an extensive data set of synthetic force traces. Proceeding down the workflow, this data set is used to train a Neural Network (NN) model able to estimate the PT model parameters from force traces. Finally, this synthetic-trained model is used to evaluate real AFM traces to extract the effective AFM parameters for the experimental system. In this study we labeled the AFM traces by an automated algorithm estimating the parameters from the traces and physical evaluations to assess the reliability of the model. (b) Colored slices show the region of relevant parameters *U*_0_ and *K* explored by the simulations. The color reports the value of dimensionless parameters η_PT_, see the colorbar on the right. Gray symbols mark the values of *U*_0_ and *K* estimated by an automated algorithm in the testing experimental data set: diamonds refer to Graphene (G)/HOPG, hexagons to G/MoS_2_, and squares to G/WS_2_. The left (tilted-line-covered) and bottom right (x-covered) patches mark the regions of smooth-sliding and large η_PT_ not relevant for the considered AFM experiments.

## Results and Discussion

The first step is to define the relevant parameter space for our data set. In a PT model suitable for simulating AFM stick–slip force traces, the key parameters are the barrier height *U*_0_ and the effective contact stiffness *K*. The mass *m* and damping coefficient γ typically assume standard values and are therefore not included in this initial analysis. The values of *U*_0_ and *K* determine the resulting dynamical regime—ranging from smooth sliding to stick–slip and multiple-slip behavior—as illustrated by the η_*PT*_ definition and examples in Figure 1b. These distinct regimes leave identifiable fingerprints in the force traces (see again Figure 1b). As shown in Figure 2b, our Langevin simulations (colored regions) span the full parameter space relevant to experimental conditions (details on how effective parameters are estimated in experiments are discussed below and in the SI). The corresponding experimental data sets for different materials are indicated by gray points, as described in the figure caption. The Langevin simulations (Sim) for a broad range of *K* and *U*_0_ values are conducted at room temperature, and with all other parameters, *m*, γ, and sliding velocity *v*_0_ fixed as detailed in the Methods and Section 1 in the SI. We focus on the stick–slip regime, including both single slip and multiple slips.^19^ This procedure yields a simulated database of 1600 trajectories to train the NN model, which we split 80/20 between training and validation.

As illustrated in Figure 3, the raw trace (force versus tip displacement) is segmented into batches, and the force values within each batch are provided as input to the neural network. The first layer of the perceptron is sized to match the length of the force input. To maintain simplicity and interpretability, the input data are unstructured, meaning that the NN is not given explicit temporal information and must infer the time-sequence nature of the data on its own. The output layer of the perceptron contains three neurons corresponding to the predicted values: the effective stiffness *K*, the barrier height *U*_0_, and the steady-state friction force ⟨*F*⟩. The first two are input parameters of the PT simulation comprising the synthetic data set: the network is shown the ground truth and is expected to learn how the force profiles relate to these parameters, effectively learning the underlying PT model. The steady-state friction force ⟨*F*⟩ is evaluated for each trajectory. In the case of experimental data, *K* and *U*_0_ are estimated from the force traces by using an ad-hoc algorithm. We therefore compare the outputs of the NN (trained on synthetic data) to the estimations from the ad-hoc algorithm, as no ground truth exists in this case. For details on the parameter space of the NN and the training, see the Methods.

**Figure 3:**
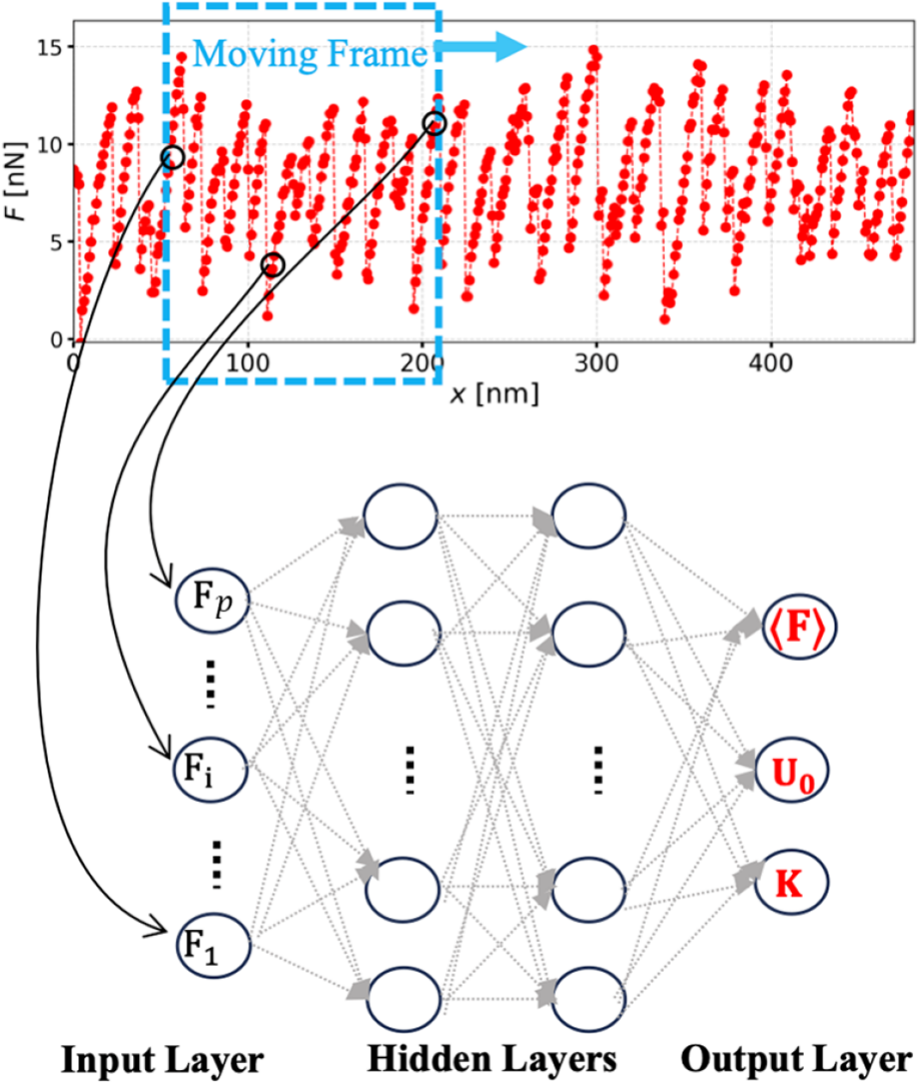
Neural network architecture and input structure. The raw force trace is divided into overlapping batches using a moving window of fixed length (top panel). Each force value within a batch is input into a separate neuron in the first layer of the neural network, as illustrated. The final layer of the multilayer perceptron outputs the predicted values: the effective stiffness *K*, the barrier height *U*_0_, and the steady-state friction force ⟨*F*⟩ (bottom panel).

The parity plots for the validation set, showing the real simulation parameters against NN predictions, are reported in Figure 4a–c. The plots correspond to the average steady-state force ⟨F ⟩, barrier height U_0_, and effective stiffness *K*, respectively. The corresponding are Root Mean Squared Errors (RMSEs) are listed in Table 1. Clearly, the NN is able to learn the parameters of the underlying model from the simulations to a near-perfect degree. This is not surprising, as the validation set is part of a coherent data set of clean data, all taken from the same statistical distribution. Moreover, the region of the parameter space from which both training and validation sets come is well sampled.

**Figure 4:**
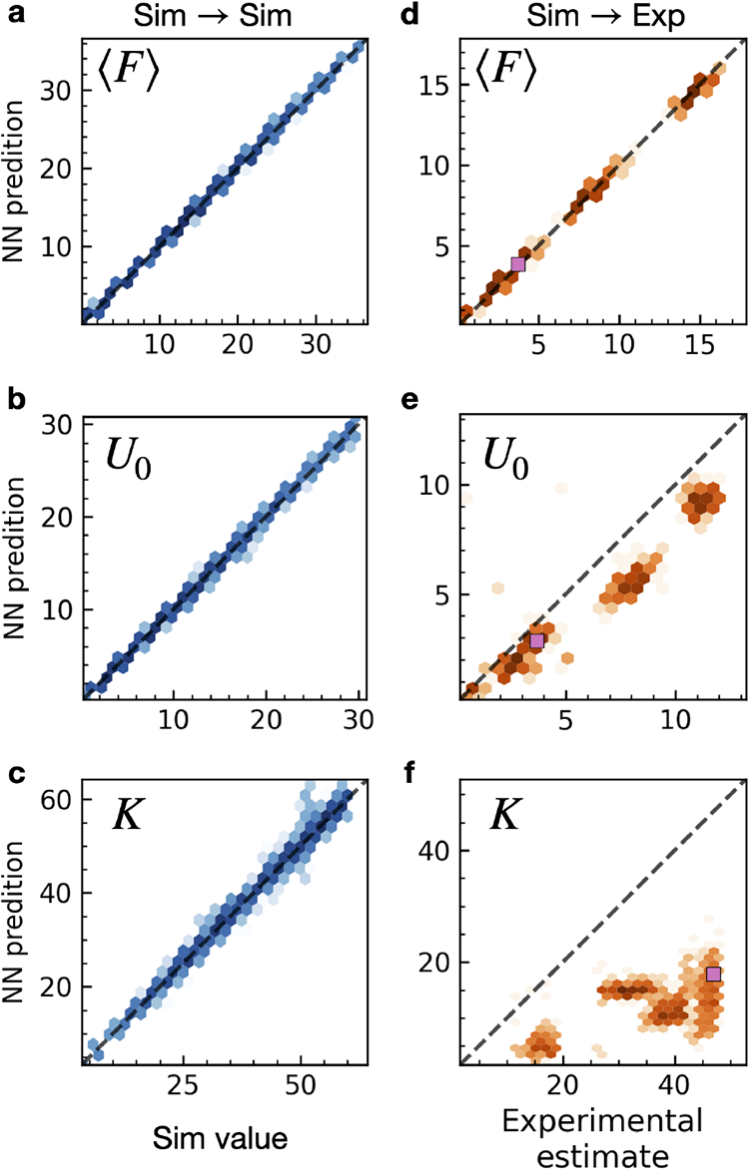
Parity plot for the NN model trained on synthetic data and used to predict other synthetic data (Sim → Sim left column of panels a–c) or experimental data (Sim → Exp right column of panels d–f). The intensity of the color reflects the local density of the points. Evaluation of the NN in predicting average force ⟨*F*⟩ (a), sliding barrier *U*_0_ (b) and effective stiffness *K* (c) of a test set of simulation that were not shown to the network during training. Evaluation of the NN in predicting average force (d), sliding barrier (e), and effective stiffness (f) of an experimental data set of which no element was ever considered during training. The pink squares mark the trajectory shown in Figure 6g and analyzed in details in Figure 6d–f.

**Table 1:** RMSE of the Same NN Model Trained on Different Dataset and Different Descriptors^a^

model/RMSE	⟨*F*⟩ [nN]	*U*_0_ [eV]	*K* [N/m]
NN Sim → Sim	0.16	0.18	0.84
NN Sim → Exp	0.31	1.78	26.62
NN Exp → Exp	0.13	0.68	2.97
NN Exp → Sim	0.42	1.54	23.33
PI-NN Sim → Exp	0.18	1.33	4.33

a NN refers to the model trained on raw force traces and PI-NN to models trained on force traces and processed derivatives. Sim and Exp refer to synthetic and experimental datasets, respectively. The one left of the arrow is the training set, and the one right of it is the evaluation set. Note that the two sets are disjoint: a NN trained on the Sim dataset is never shown an Exp force trace during training.

An instructive benchmark is to compare the performance of the neural network on synthetic data—where the true values of the barrier height *U*_0_ and effective stiffness *K* are known—with that of an ad hoc algorithm specifically developed to analyze AFM force traces of this type. It is important to emphasize that stick–slip force traces are inherently complex, and traditional fitting procedures often rely heavily on prior knowledge to identify which features to include and which to disregard, typically restricting the analysis to single-slip events. In contrast, our ML-based approach requires no such assumptions, making it significantly more robust and user-friendly.

These advantages are illustrated in detail in Section 3 in the SI, where we present a direct comparison between the neural network and the ad-hoc algorithm on the synthetic data set. The neural network not only outperforms the traditional algorithm in terms of accuracy but, more importantly, demonstrates a substantially wider range of applicability.

To assess whether the physical contents embedded in the model trained on synthetic data can be recognized in actual frictional stick–slip force traces, we submit to the NN our own data set of 1298 AFM experimental trajectories, akin to the one shown at the bottom of Figure 1a. The general setup of an AFM experimental system is sketched in Figure 1a. While in the rest of the work we will deal with our own realization of the AFM experiments involving colloidal probes, we note that no assumption linked to the specific setup are made in our treatment and, thus, we believe our results are relevant for any AFM experiment observing atomic stick–slip.^20–29^ Our AFM colloidal tip is capped with a nanorough graphene coating of mesoscopic size, which ultimately controls the contact mechanics and friction through tens-of-nanometers tall protrusions that slide at ambient conditions on the substrate. Substrates were either graphite (HOPG) or TMDs with a crystalline axis parallel to the tip sliding direction.^14,30,31^ Unlike the perfect sketch of Figure 1a, the contact itself will inevitably contain structural defects and adsorbates (see Methods and Section 2 in the SI for details). The putative PT parameters that best describe the experimental force traces in our experimental setup were in fact estimated manually in previous work, with an ad hoc algorithm.^15,30,32,33^ That and other works indeed suggested that the PT model, generally used for point-like sliders, could capture well the frictional physics of sliding contacts up to mesoscopic size.^3,13^

As reported in Figure 4d–f, the learning of average friction ⟨*F*⟩ and barrier *U*_0_ extrapolates well from synthetic trajectories to experimental ones, while the extrapolation of *K* fails: the predicted values are scattered almost randomly. What is the origin of this failure? First, we did a necessary sanity check. The culprit is not simply the fact that the experimental traces do not carry enough information for the NN model to learn. Indeed, training the model on experimental trajectories can relatively well predict the parameters of other experimental trajectories; see SI Section 4 and Table 1. Moreover, the fault lies not with the ad hoc algorithm either. If this algorithm is used to estimate the PT parameter from synthetic traces, one recovers the known input parameter with reasonable accuracy (see SI Figure S3c,d). The small deviations actually resemble those one would find if the parameters *K* and *U*_0_ were hypothetically extracted by hand from the simulated force traces—the latter an operation that makes no further sense in our context.

In fact, the step we are attempting is less trivial than one may suppose from the similarities between force traces in Figure 1. The synthetic and experimental force traces originate from different systems and are thus drawn from different statistical distributions. As the training is done only on the synthetic trajectories, the NN is not penalized for predicting poorly the effective parameters that best describe the experimental force traces. When the network is trained only on synthetic data, it may in fact become overly specialized in recognizing features unique to this data set rather than more general, physics-based fingerprints in the traces. Learning such general features would permit a more accurate extraction of the best effective PT parameters from the experimental force traces.

In order to test this hypothesis, we mixed a fraction of the experimental trajectories in the training set. As shown in SI Section 4.1, the addition of a small fraction of 1% of experimental data in the training set helps the NN to generalize better. As it exposes the model to the experimental data distribution, with its real-world variability and noise, the mixed data set encourages the model to focus on general, physics-based features rather than features specific to the synthetic data. Note that the reverse reasoning works as well: a model trained solely on experimental data fails to predict the synthetic data correctly, see SI Section 4 and Table 1.

That said, we still need to make progress on a scheme where the training is restricted exclusively to synthetic data. As we shall see in the following section, it is possible to enhance the perception of physically meaningful parameters by elaborating the synthetic training data in a way that nudges the NN’s learning process in the right direction.

### Physics-Informed Data Augmentation

We can use our own knowledge of how the physics of the PT model reflects the force traces to guide the NN training. We start by noticing that the NN can predict well the barrier height *U*_0_, see Figure 4e. Consider the synthetic and experimental force traces reported in Figure 5.

**Figure 5:**
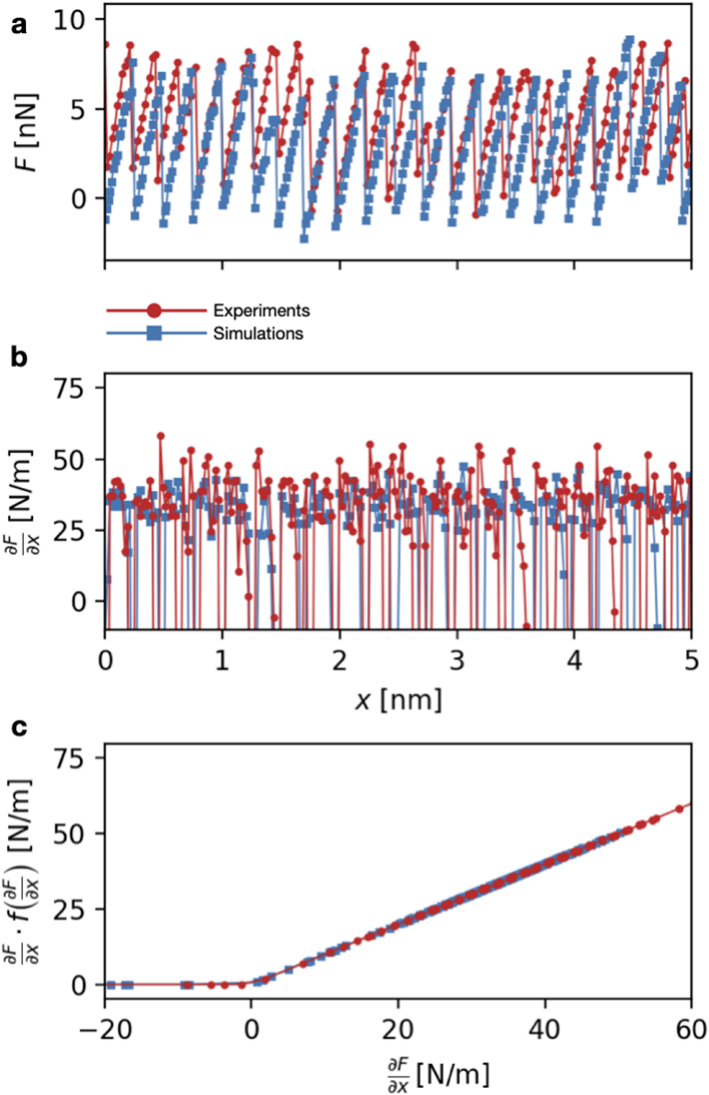
(a) Example of force traces fed to the NN during training. Red and blue symbols refer to an experimental and simulated trace (respectively) with similar parameters. (b) Derivative of the force traces in panel a. (c) Processed derivative fed to NN during training.

The barrier is a “global” quantity in the force trace, proportional to the maximum of the recorded force^15,30,32^ (see SI Section 2). On the other hand, the effective stiffness *K* is a more “local” quantity: to estimate it from the force trace, one has to consider the slope of the trace in the “sticking” part, when the spring charges before the mechanical slip instability happens.

The stiffness is thus a function of the force derivative *K* = *K*(∂*F*/∂*x*). In order to nudge the learning of the NN model in this direction, we therefore feed to the model, in addition to the force trace, also its derivative ∂*F*/∂*x*, computed through finite differences, see Figure 5b. We note in addition that only the sticking portion of the trace, where the force slope is positive, is relevant to stiffness, while negative slopes is not and could be set to zero in the NN training. Considering that in experiments the slopes at the sticking parts tend to be smaller than the real maximum slope (Figure 4a) due to multiple factors (e.g., defects, temperature, sliding directions), one may strategically reduce the influence of these smaller slopes on the NN by decreasing their weight. To achieve this, the derivative is weighted with the sigmoid function *f*(*x*) = 1/(1 + exp­(−(*x* – μ)/σ)), where μ is the mean and σ = 15 N/m is a smearing parameter. As our simple network has no positional encoding, we simply concatenate the function and its processed derivative as an input. Hence, we feed the NN the sorted value of this function, ∂*F*/∂*x*·*f*(∂*F*/∂*x*), yielding the curves reported in Figure 5c. The proposed augmentation is grounded in the physical principles of the PT model: it is not merely an empirical weighting function. By suppressing the negative peaks corresponding to “slip” events, the neural network is guided to focus on the “stick” phases, where, based on physical insight, the most relevant information is encoded. This approach leverages our understanding of atomic friction dynamics to enhance the effectiveness of the learning process.

This physics-driven preprocessing step improves the ability of the NN to learn the physical principles that underlie the role of *K* in shaping the force trace and ensures a better generalization to experimental data.

The results of this new procedure are reported in Figure 6a–c. Note that now the NN, trained solely on synthetic data, is able to make a reasonable prediction for all the relevant experimental parameters, including the spring constant *K*, see also Table 1. This is remarkable as the network has never seen any experimental trace. We interpret this as evidence that the NN has learned the key physical mechanism rather than a database-specific pattern and, thus, is able to extrapolate from synthetic traces to experimental ones, of course, within the limit of our crude approximations and model, which justifies the spread in the predicted points. A separate origin for the spread is the defects and variability in the experimental samples; indeed, training a NN on experimental data to predict other experimental data results in a similar spread (see SI Section 4 and Table 1), hinting at the higher noise intrinsic to the experimental data set.

**Figure 6:**
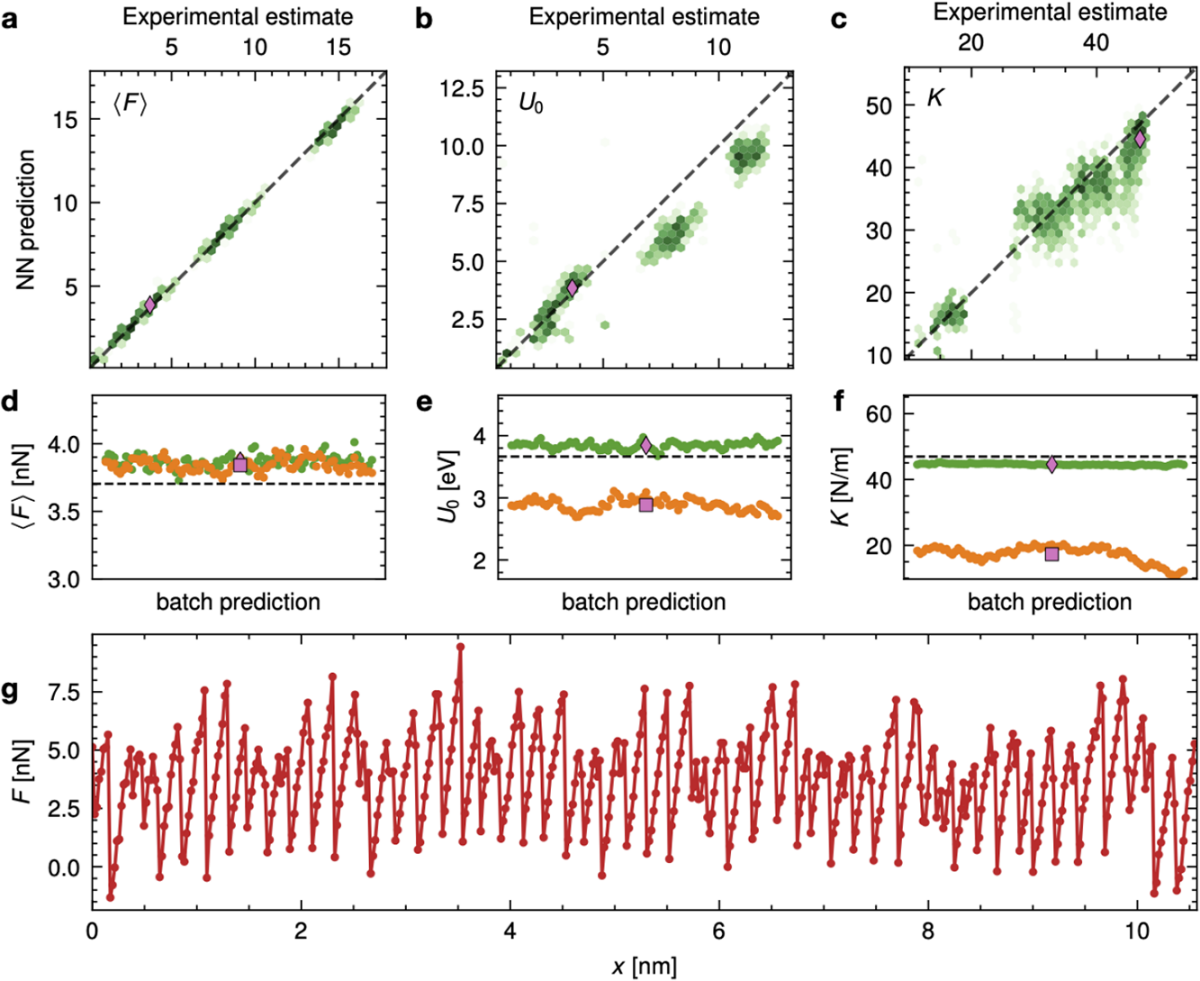
NN trained on physics-augmented synthetic data and evaluated on experimental data. The color intensity reflects the local density of points. The NN prediction for (a) average force ⟨*F*⟩, (b) sliding barrier *U*_0_ and (c) effective stiffness *K* of an experimental data set of which no element was ever considered during training. Note that the network has now learned to predict *K* for the experimental data, albeit with a widespread. The pink diamonds mark the trajectory shown in panel g and analyzed in details in the panels d–f. Batch estimates for (d) ⟨*F*⟩, (e) *U*_0_, and (f) *K* from the bare NN (orange points) and physics-embedded NN (green points) for the experimental force trace reported in panel (g) (red points). This experimental force trace corresponds to the pink diamond in panels a–c and to the pink square in Figure 4d–f. The dashed lines indicate reference values obtained from experimental data using the automated algorithm outlined in SI Section 2. The averaged prediction for the physics-embedded NN (pink diamond) agrees with the estimated value, while the bare NN’s prediction is largely underestimated, and the batch-wise estimate shows a nontrivial scattering, suggesting that the bare NN has not properly learned how to accurately evaluate *K*.

The augmented synthetic training scheme, now including the rectified force derivative, outperforms the one trained on raw traces in all predictions, as shown in Table 1. The batch-wise predictions for the specific experimental trajectory in Figure 6g (highlighted by the pink symbols in Figures 4d–f and 5a–c) are shown in Figure 6d–f. Comparing cases where predictions for both are poor, these occur when the experimental traces are noisy and irregular, suggesting that the AFM tip is likely crossing structural defects and different domains. In such cases, even a human estimate of the PT parameters, our reference label here, is not without ambiguity. Moreover, as *U*_0_ decreases and *K* increases, the system approaches the smooth sliding regime. The stick–slip fingerprint weakens, and thus, estimating the system parameters from it becomes less and less reliable, becoming totally impossible in a regime of smooth sliding.

As a last observation, part of the uncertainty of the parameters *K* and *U*_0_ can be instructively explained on physical grounds. The PT model stiffness *K* is that of an ideal elastic spring. The effective experimental contact stiffness is considerably more complicated, generally involving dissipative and plastic deformations taking place under practical loading conditions. As stress builds up toward the end of a sticking interval, the real contact undergoes precursor relaxations before yielding, thus softening the friction force slope just prior to slip. The NN reads that last-minute softening as a decrease of overall stiffness and barrier below those far from the slip.

Besides that precursor softening, the small systematic underestimation of the barrier *U*_0_ also depends on our assumption of a graphene lattice spacing in all simulations, whereas the actual experimental input was more varied, including materials with a lattice spacing slightly larger than that of graphene. The systematic underestimate of *U*_0_ is a perfectly reasonable result rooted in that approximation—necessary in this conceptual work—rather than NN capabilities (see SI Figure S6b,n). In fact, this result highlights the considerable robustness of our ML approach.

## Conclusions

We have shown that NN can learn nanoscale friction within the framework of the PT model. In order to extrapolate from synthetic, simulated data to real experimental data, care must be exercised: a model too specialized to a single-origin data set may fail spectacularly. To prevent this, the NN training should be constructed to include enough physically relevant descriptors. In this exploratory work, we limited ourselves to a relatively simple NN architecture according to the fundamental character of our investigation. Our aim was to show that indeed, the PT framework can be learned by an NN model. As a result, we confirm that training on strictly synthetic data can indeed yield reasonable predictions for the best parameters that physically describe completely unknown experimental data. The ML scheme can be naturally extended, if desired, to involve sliding models richer than simple PT, with new parameters related to other local features of the force traces that could be learned, possibly by additional suitable augmentations of the synthetic training data, as was done here for stiffness. We also believe that deploying a more sophisticated ML model exploiting the sequential nature of the data, such as a recurrent neural network or even an attention-based model, could improve learning accuracy. The scheme outlined in this work should prove of considerable practical use in direct connection with experimental AFM data postprocessing, as well as of physical value in their interpretation.

## Methods

### Synthetic Friction Simulations

A fourth-order Runge–Kutta algorithm was used to propagate the (underdamped) Langevin equation in eq 4 in SI Section 1.^34^ The instantaneous lateral force trace was evaluated as *F* = −*K*(*x* – *v*_0_ *t*) to obtain friction force vs cantilever displacement traces. We used a tip mass of *m* = 1 × 10^–12^ kg. The Langevin damping coefficient was set to 2γ*m* = 0.01 ns^–1^ to match the experimental force traces' amplitude. To simulate experimental conditions, we selected a sliding velocity of *v*_0_ = 60 nm/s and a temperature of *T* = 296 K. The parameter space was sampled in the relevant region (0.2 < *U*_0_ < 25 eV, 3 < *K* < 60 N/m with the constraint η_PT_ < 30) according to experimental estimates (see Figure 2 in the main text) with 1600 randomly distributed points, each corresponding to a steady-state simulation. To be consistent between synthetic and experimental data sets, the simulated force traces were linearly interpolated to match the displacements imposed by the experimental lower sampling frequency: each simulated force trace was reduced to 512 points in which the moving support traveled the same distance as the cantilever in experiments.

### Experimental Setup

The atomic-scale friction force spectroscopies were carried out under standard laboratory conditions by means of a commercial AFM operated in contact mode (Solver P47-PRO by NT-MDT, Russia). An additive-free aqueous dispersion of graphene was used to prepare graphene-coated colloidal probes (based on silica beads of ∼25 μm diameter), following a fabrication method ”mixing” dip-coating and drop-casting techniques.^14^ High-resolution micrographs revealed that the graphene coating was generally inhomogeneous at the submicrometric scale, with uncoated silica regions interspersed with tens-of-nanometers tall protrusions formed by randomly stacked and/or highly crumpled flakes. Despite their random accumulation and nonconformal adhesion to the silica surface, the agglomerated flakes maintained a lubricious behavior.^14^ Consequently, the manifestation of graphene-mediated friction effects was ultimately controlled by the topographically highest contact nanoasperity.^30,31^ The graphene-coated colloidal probes were placed in contact with the freshly cleaved surfaces of HOPG (grade ZYB by MikroMasch), 2H-WS_2_ or 2H-MoS_2_ crystals (from HQ Graphene), respectively, thus leading to the realization of different sliding interfaces between 2D materials (as shown in Figure 1a in the main text). For the calibration of the elastic constant of each probe *k*_C_, and of the normal force *F*_N_ and lateral force *F*_L_, see the Supporting Information Section S1 in refs 14 and 33 . We obtained load-dependent atomic-scale stick–slip trajectories from friction maps (512 × 512 pixels), in which *F*_N_ was systematically decreased every ten lines from a relatively large starting value (i.e., a few hundreds of nN) to the pull-off point.

We interrogated surface portions that were free from atomic steps, with a typical scan range of 11 × 11 nm^2^ and sliding velocity *v*_0_ ∼ 30 nm/s. Representative stick–slip trajectories for each system are shown in Figure S2 in the SI. The data set of 1298 AFM experimental trajectories comprised: 459 traces for the graphene/HOPG interface; 429 traces for the graphene/MoS_2_ interface; 410 traces for the graphene/WS_2_ interface.

### Neural Network Model

This investigation leverages PyTorch^35^ to manage the data set and train a multilayer perceptron (MLP) architecture designed to predict multiple target features related to friction data, including average force ⟨*F*⟩, sliding barrier *U*_0_, and effective stiffness *K*. The input to the network is a fixed number *p* = sample_size of consecutive samples from the original force trace. For the physics-informed model, we concatenate the trace and finite element processed derivative for each sample, resulting in an input dimension *p* = sample_size × 2 – 1. The MLP consists of two hidden layers with 256 and 128 neurons, respectively, utilizing the Rectified Linear Unit (ReLU) activation function to introduce nonlinearity and enhance the model’s ability to capture complex patterns. The output layer is linear and has a size equal to the number of target quantities. The loss function is the sum of the quadratic loss for each target. The model is trained over 50 epochs with a batch size of 300, using the Adam optimizer with a learning rate of 5 × 10^–4^.

## Supplementary Material





## Notes

Details of the augmented PT model, experimental setup details, ad hoc algorithm, additional NN benchmark and details (PDF)
Example file: synthetic dataset, ML source code and Jupyter notebook with NN training and evaluation (ZIP)
