# Supplementary material for: Can Neural Networks Learn Atomic Stick–Slip Friction?
Source: ACS Appl Mater Interfaces. 2025 Jul 9;17(29):42454–61. doi: 10.1021/acsami.5c09866 (PMC12291085; doi:10.1021/acsami.5c09866)
Supplement: Supplementary file 1 [file am5c09866_si_001.pdf]

Supporting Information for Can Neural Networks Learn Atomic Stick-Slip Friction?

Mahboubeh Shabani,[†,⊥] Andrea Silva,[*,‡,∥] Franco Pellegrini,[¶] Jin Wang,[¶,⊥] Renato Buzio,[§] Andrea Gerbi,[§] Andrea Vanossi,[‡,∥] Ali Sadeghi,[†] and Erio Tosatti[*,¶,#]

†*Department of Physics, Shahid Beheshti University, Tehran 1983969411, Iran*

‡*CNR-IOM, Consiglio Nazionale delle Ricerche - Istituto Officina dei Materiali, c/o SISSA, Via Bonomea 265, 34136 Trieste, Italy*

¶*International School for Advanced Studies (SISSA), Via Bonomea 265, 34136 Trieste, Italy*

§*CNR-SPIN, C.so F.M. Perrone 24, 16152, Genova, Italy*

∥*International School for Advanced Studies (SISSA), Via Bonomea 265, 34136 Trieste, Italy*

⊥*International Centre for Theoretical Physics (ICTP), Strada Costiera 11,34151 Trieste,Italy*

#*CNR-IOM, Consiglio Nazionale delle Ricerche - Istituto Officina dei Materiali, c/o SISSA, Via Bonomea 265, 34136 Trieste, Italy*

E-mail: silva@iom.cnr.it; tosatti@sissa.it

1 Details of PT model

In the standard Prandtl-Tomlinson (PT) model, the total potential energy experienced by the tip can be written as:

$$U(x,t) = -\frac{U_0}{2}\cos\left(\frac{2\pi}{a}x + \pi\right) + \frac{1}{2}K(v_0 t - x)^2. \tag{1}$$

The first term describes the corrugation potential (tip-substrate interaction) where U_0 is the barrier amplitude of the corrugated substrate, x is the tip displacement, and a is the lattice spacing of the substrate. The second term is elastic interaction between the tip and the moving support. The effective spring constant is denoted by K. Note that K is not only due to the torsional stiffness of the cantilever but also includes the contribution from the lateral stiffness of the contact and the details of the tip structure.

We used a tip mass of $m = 1 \times 10^{-12}$ Kg. The Langevin damping coefficient was set to $2\gamma m = 0.01$ ns^{-1} to match experimental force traces amplitude. To simulate experimental conditions, we selected a sliding velocity of $v_0 = 60$ nm/s and a temperature of $T = 296$ K.

The augmented PT model utilized in this study incorporates a standard sinusoidal modulation of height U_0 and spacing a, enhanced by a localized distortion that mimics defects found on real surfaces. This distortion is achieved through the convolution of an array of Gaussian functions with an additional sinusoidal potential, both incommensurate with the periodicity of the original substrate.

A Gaussian-shaped, long wavelength modulation is combined with another sinusoidal substrate, incommensurate to the standard one, as shown in Figure S1. The resulting perturbation has the form

$$U'(x) = \epsilon\frac{U_0}{2}\cos\left(\frac{2\pi}{c}x + \pi\right)\exp\left(-\frac{1}{2}\left(\frac{g(x)}{\sigma^2}\right)^2\right) \tag{2}$$

$$g(x) = \cos\left(\frac{2\pi}{d}x\right), \tag{3}$$

where $\epsilon = 0.7$ is the ration between amplitude of the standard PT susbstrate and the perturbation, $c = 31.415\,\mathrm{pm}$ and $d = 619.3\,\mathrm{pm} \approx 25b$ are the spacing of the second sinusoidal and gaussian potentials, respectively, and $\sigma = 0.2$ is the smearing of the Gaussian modulation. The parameters of the perturbation were kept fixed in the whole study and the perturbation potential itself is added to the total energy in Eq. (1).

The dynamic of tip is described by the following Langevin equation:

$$m\ddot{x} = -\gamma\dot{x} - \frac{\partial U(x,t)}{\partial x} + \xi(t) \tag{4}$$

where m is the effective mass of tip, γ is the damping coefficient, t is time. The thermal noise term $\xi(t)$ satisfies the fluctuation-dissipation theorem: $\langle \xi(t)\xi(t') \rangle = 2m\gamma k_B T \delta(t - t')$.

The PT model predicts two distinct regimes for the tip motion, depending on the dimensionless PT parameter:[1]

$$\eta_{PT} = \frac{4\pi^2 U_0}{ka^2}, \tag{5}$$

which represents the ratio between the stiffness of the tip-substrate potential and the pulling spring. When $\eta_{PT} < 1$, the time-dependent potential U exhibits only one minimum and the sliding motion is smooth; for $\eta_{PT} > 1$, two or more minima appear in U, and the sliding is discontinuous, characterized by stick-slip events.

2 Experimental details and analysis of experimental trajectories by means of an automated algorithm

To validate the NN predictions for the experimental datasets, we independently processed the raw force traces with an automated algorithm. This algorithm analyzes the individual stick-slip events after identifying them through thresholding criteria.[2,3] The algorithm was implemented in LabView (National Instruments) and provided estimates for the barrier height U_0, the effective lateral stiffness K and the Tomlinson parameter η_{PT} as a function of

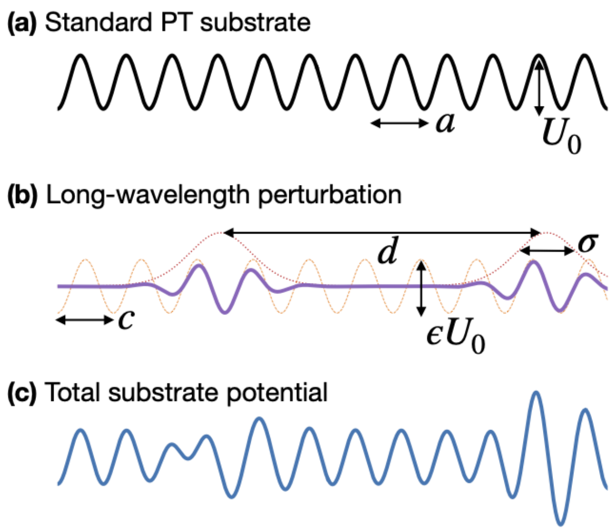

Figure S1: Substrate potential in the augmented PT model. The standard PT substrate in panel (a) is combined with a long-wavelength localized perturbation in panel (b) to yield the quasi-periodic potential in panel (c).

the normal load F_N. Briefly, for each value of F_N, we selected only those specific portions of the experimental lateral force traces having periodicity a (0.21-0.25 nm for HOPG; 0.29-0.32 nm for WS_2 and MoS_2). In fact these correspond to individual slip jumps of the AFM probe approximately along the zigzag crystallographic direction. Smaller (or longer) slips than a were disregarded. For each experimental force trace, the corrugation U_0, the contact stiffness K, and the PT parameter η_{PT} were estimated as:

$$U_0 = \frac{aF_{L,max}}{\pi} \tag{6}$$

$$\eta_{PT} = \frac{2\pi F_{L,max}}{ak_{exp}} - 1 \tag{7}$$

$$K = \frac{\eta_{PT}+1}{\eta_{PT}}k_{exp} \tag{8}$$

where $F_{L,max}$, a and k_{exp} are the highest local force maxima, the slip distance, and the lateral force slope respectively. To designate the highest force maxima along any selected force trace, we ordered the jumps of slip distance a in terms of decreasing force amplitude F_L

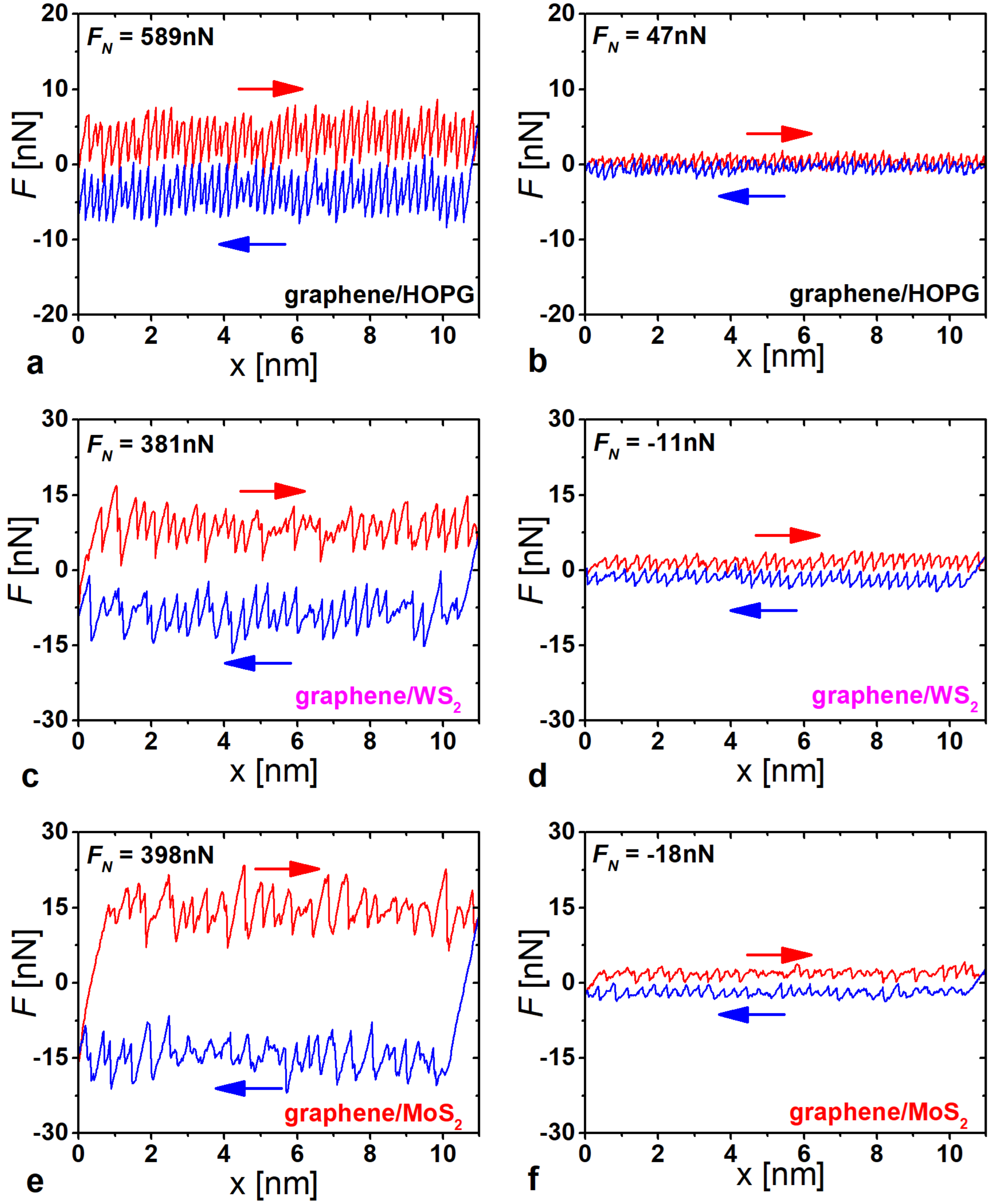

Figure S2: Representative load-dependent friction loops acquired on HOPG ((a),(b)), 2H-WS_2 ((c),(d)) and 2H-MoS_2 ((e),(f)) by means of graphene-coated colloidal probes.

and we conventionally assumed $F_{L,max}$ to correspond to the 25% tail of the highest jumps. For each normal load value F_N, the mean values of U_0, K and η_{PT} were obtained by averaging over an ensemble of several slip jumps, as shown in Figure S3.

We preliminarily validated the automated algorithm by processing a set of 840 synthetic force traces, attesting good accuracy (7-10%) in the estimation of the contact parameters U_0, K only for those force traces characterized by prominent stick-slip instabilities. At room temperature, this corresponds to the condition $\eta_{PT} \gtrsim 2$.[4]

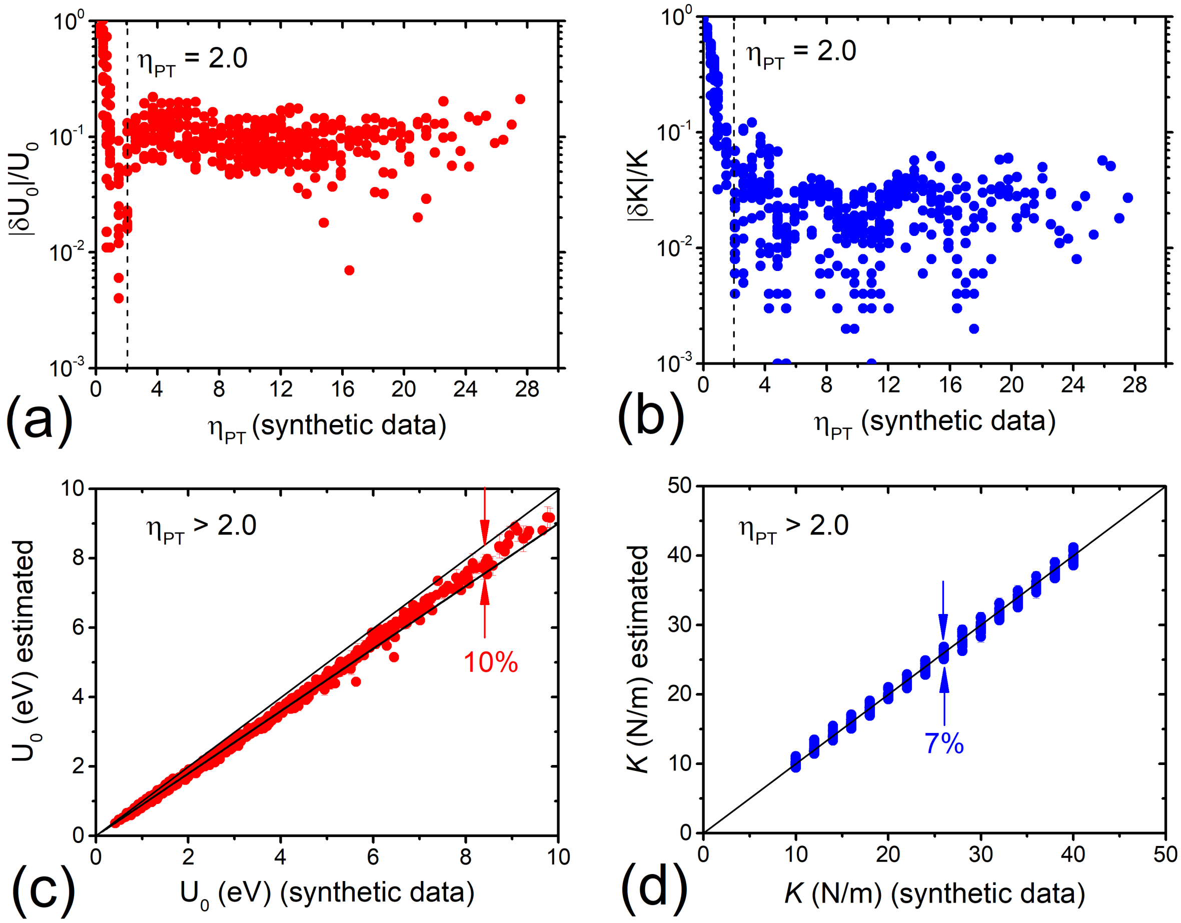

Figure S3: Validation of the automated algorithm by processing a set of 840 synthetic force traces. (a),(b) Variation of the relative error for the contact parameters U_0 and K against the η_{PT} parameter. (c) and (d) are the parity plots quantifying the algorithm accuracy to estimate U_0 and K respectively. Note in the latter panels the good accuracy with which these PT input parameters, in abscissa, are predicted by the automated algorithm, in ordinates. The accuracy is higher for the stiffness than for the barrier, whose value is only slightly underestimated.

Figure S4 shows the contact parameters U_0 and K we estimated with the automated algorithm for the AFM experiments discussed in the main text.

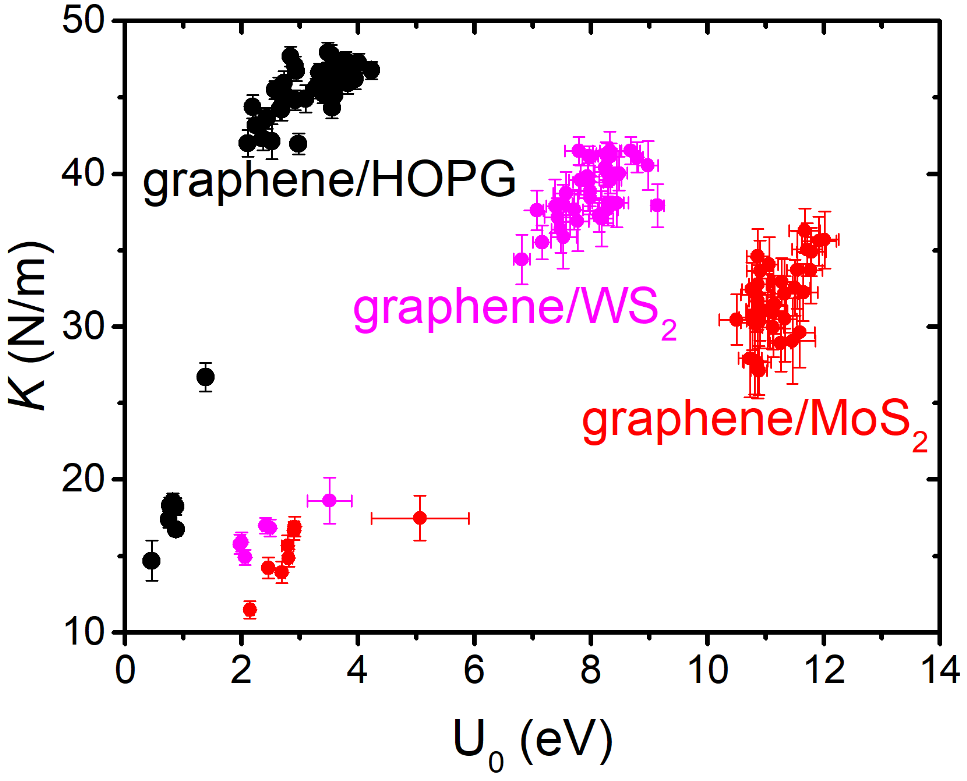

Figure S4: Contact parameters U_0 and K estimated from experimental stick-slip trajectories using the automated algorithm.

3 Comparing NN and ad-hoc algorithm

We benchmark our neural network against a purpose-built algorithm developed specifically for these systems (Refs[4–6]). It is important to note that are complex, and Traditional fitting methods for stick-slip force traces depend heavily on prior knowledge of which features to fit and which to disregard. For instance, the approach in Ref[4] performs fitting by arbitrarily restricting the analysis to specific regions of single-slip events, ignoring multiple slips and other patterns deemed less representative. In the absence of such prior knowledge—which is often unavailable in general cases—traditional fitting becomes significantly more difficult, if

not unfeasible.

In contrast, our ML-based approach does not rely on such assumptions: it learn how to correlate features from the full force traces to the observed values. This unbiased approach makes it more robust and user-friendly.

Table S1: Comparison of RMSE values for K and U_0, and $\langle F \rangle$, between the neural network (NN) and the ad-hoc algorithm.

	**NN**	**ad-hoc algorithm**
RMSE K [N/m]	0.24	1.46
RMSE U_0 [eV]	0.13	0.40
RMSE $\langle F \rangle$ [nN]	0.14	0.25

Figure S5 and Table S1 present the comparison between the neural network and the ad-hoc algorithm on a synthetic dataset. The neural network outperforms the ad-hoc algorithm overall, as indicated by the lower RMSE values in Table S1. More importantly, the plots highlight that the neural network has a much broader range of applicability. For example, see Figure S5 in cases with $\eta_{PT} < 2$ (green crosses), the ad-hoc algorithm is less accurate. Critically, when dealing with trajectories that include only multiple slips (black crosses), the algorithm fails entirely. This clearly demonstrates that our neural network approach not only performs better in terms of accuracy but also significantly expands the range of conditions under which reliable analysis is possible.

4 Training Neural network on experimental data

We conducted a sanity check to confirm that the experimental traces contained sufficient information for the NN model to learn.

Figure S6a-c shows the parity plot for a NN trained on the experimental dataset predicting other experimental data. Note that while the NN is clearly learning how to predict U_0 and K within this dataset, the performance is substantially worse than for the Sim $\rightarrow$ Sim case reported in Figure 4a-c in the main text. This reflects the more complex nature and

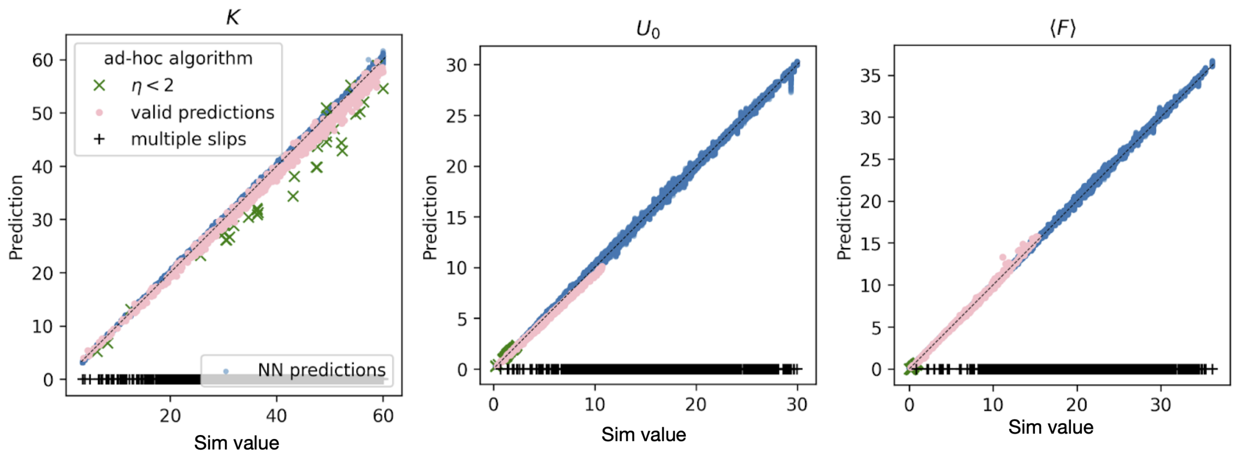

Figure S5: Parity plots for the predictions of K, U_0 and $\langle F \rangle$ from raw synthetic forces traces obtained with the NN (blue dots) and with the ad-hoc algorithm. For the latter, pink dots refer to valid predictions (single slip trajectories with $\eta_{PT} > 2$), green crosses to prediction outside the intended range ($\eta_{PT} < 2$) and black crosses to failed predictions (trajectories with only multiple slips).

variability intrinsic to the experimental data.

Figure S6d-f shows the parity plot for a NN trained on the experimental dataset predicting synthetic data. The NN fails to predict the stiffness K in a previously unseen dataset, alike the failure of Sim→Exp in in Figure4 d-f in the main text. Again, without any penalty for poor prediction of this other dataset or any physics-based descriptor, the NN generalises poorly.

4.1 Including a fraction of experimental data in synthetic database

To check that the model's predictive capabilities across datasers could be improved, we augmented the synthetic dataset with a just one percent of experimental traces. Figure S6g-m shows the parity plot for a NN trained on the hybrid synthetic data predicting Experimental data. The results demonstrating the enhanced K-prediction capability of the neural network model trained on a hybrid dataset, thereby confirming the valuable physical information contained within the experimental traces. This minimal addition significantly improved the neural network's accuracy in predicting K, demonstrating the NN's ability to learn physically

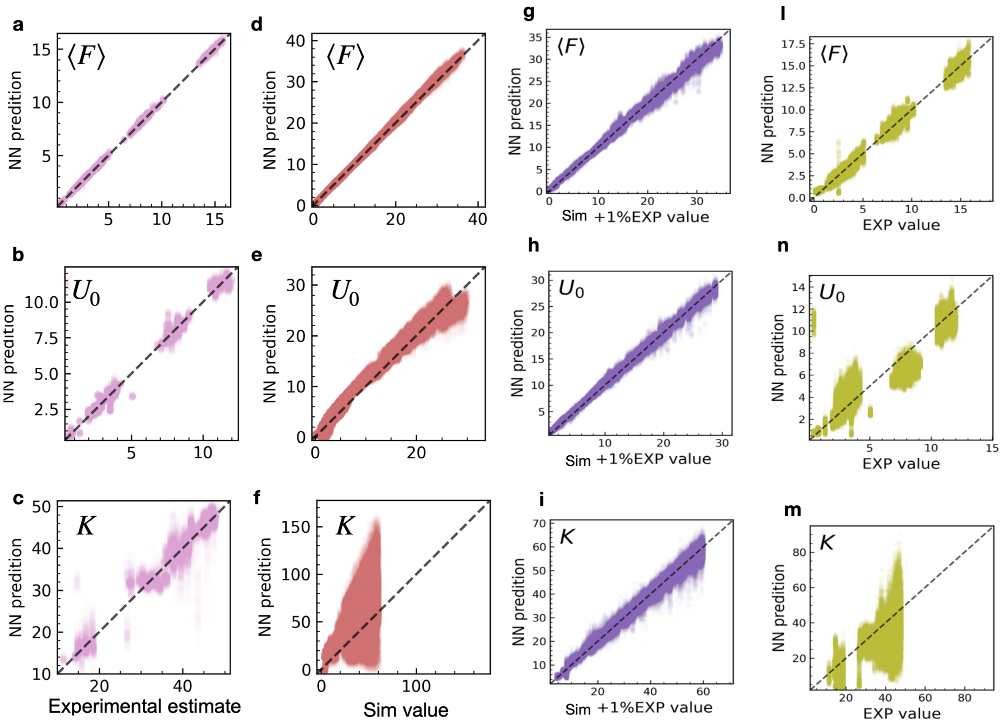

Figure S6: Parity plot for the NN model trained on experiment data. Evaluation of the NN in predicting average force $\langle F\rangle$ (a), sliding barrier U_0 (b) and effective stiffness K (c) of a test set of experiment that were not shown to the network during training. (d,e,f) Evaluation of the experimental-trained NN on the synthetic dataset. (g-m) Performance of the NN model trained on hybrid dataset (0.99 synthetic data and 0.01 of experimental data): (g-i) parity plots on the training set, (l-m) parity plots for the testing set (0.01 synthetic data and 0.99 experimental data)

meaningful features beyond dataset-specific ones.

References

1. Medyanik, S. N.; Liu, W. K.; Sung, I.-H.; Carpick, R. W. Predictions and Observations of Multiple Slip Modes in Atomic-Scale Friction. *Phys. Rev. Lett.* **2006**, *97*, 136106.

2. Schirmeisen, A.; Jansen, L.; Fuchs, H. Tip-jump statistics of stick-slip friction. *Phys. Rev. B* **2005**, *71*, 245403.

3. Jinesh, K. B.; Krylov, S. Y.; Valk, H.; Dienwiebel, M.; Frenken, J. W. M. Thermolubricity

in atomic-scale friction. *Phys. Rev. B* **2008**, *78*, 155440.

4. Buzio, R.; Gerbi, A.; Bernini, C.; Repetto, L.; Silva, A.; Vanossi, A. Dissipation Mechanisms and Superlubricity in Solid Lubrication by Wet-Transferred Solution-Processed Graphene Flakes: Implications for Micro Electromechanical Devices. *ACS Appl. Nano Mater.* **2023**, *6*, 11443–11454.

5. Buzio, R.; Gerbi, A.; Bernini, C.; Repetto, L.; Vanossi, A. Sliding Friction and Superlubricity of Colloidal AFM Probes Coated by Tribo-Induced Graphitic Transfer Layers. *Langmuir* **2022**, *38*, 12570–12580.

6. Buzio, R.; Gerbi, A.; Bernini, C.; Repetto, L.; Vanossi, A. Graphite superlubricity enabled by triboinduced nanocontacts. *Carbon* **2021**, *184*, 875–890.
